# The Habitat of the Neglected Independent Protonemal Stage of *Buxbaumia viridis*

**DOI:** 10.3390/plants10010083

**Published:** 2021-01-02

**Authors:** Ameline Guillet, Vincent Hugonnot, Florine Pépin

**Affiliations:** 1 Independent Researcher, 58 rue Georges Rissler, FR-63000 Clermont-Ferrand, France; guillet.ameline@gmail.com; 2 Independent Researchers, Le Bourg, FR-43380 Blassac, France; flopepin@gmail.com

**Keywords:** gemmae, decaying wood, dead-wood, ecological modelling, conservation

## Abstract

*Buxbaumia viridis* is a well-known species of decaying deadwood, which is protected in Europe. All previous studies dealing with the ecology of *B. viridis* rely on the sporophyte generation because the gametophyte generation is allegedly undetectable. Recent advances have shown that the protonemal stage, including gemmae, is recognizable in the field, thereby considerably modifying our perception of the species’ range and habitat. In France, we demonstrate the existence of independent protonemal populations, with the implication that the range of *B. viridis* is widely underestimated. Sporophytes and sterile protonema do not share the same ecological requirements. The sporophyte stage was found in montane zones, almost exclusively in coniferous forests, and on well-decayed wood. The sterile protonemal stage extends to lower elevations, in broad-leaved forests, and on wood in a less advanced state of decay. Our results suggest that the humidity could be one of the most relevant explanatory variables for the occurrence of sporophytes. Opening of the canopy seems to promote sporophyte development. Previous anomalous observations of *B. viridis* growing on humus or bark might be explained by the presence of a protonemal population that is able to produce sporophytes under rarely occurring but favorable climatic events.

## 1. Introduction

*Buxbaumia viridis* (Moug. ex Lam. et DC.) Brid. ex Moug. et Nestl. (Buxbaumiaceae) is a hygrophilous, sciaphilous, and acidophilous species, widely distributed in the Northern hemisphere, where it mainly colonizes decaying wood in wet, shaded coniferous montane woodlands [1,2,3]. Ecological factors, that have been suggested as relevant in explaining the occurrence of the species, are the amount of available deadwood, the degree of its decomposition, canopy openness, humidity, elevation, aspect, and deadwood composition [3,4,5]. As a species apparently linked to well-preserved forest stands with large accumulations of bulky woody debris, it has been suggested that *B. viridis* is an indicator of ancient woodland [5]. 

*B. viridis* is protected in Europe by Annex I of the Bern Convention [6], and Annex II of the “Habitats-Fauna-Flora” Directive (Habitat Directive 92/43/EEC), since a large proportion of its habitat has disappeared worldwide during the 20th century [7]. Over recent decades the species has been assessed in the European Red-list for bryophytes as “vulnerable”, and this has led to a disproportionate increase in the number of targeted studies [3,4,8,9]. Hence its status has changed to “least concern” in the most recent Red-List [10]. However, its conservation remains a matter of critical concern, for instance on account of forest management practices that lead to a reduction in coarse wood debris (CWD), or to clear-felling which permits light penetration and consequently drought [11,12].

Unlike other mosses whose haploid gametophyte is dominant and clearly visible, *B. viridis* possesses a minute gametophyte which consists of a filamentous protonema that produces much reduced female and male buds [4,13]. The distinctive protonema has been previously described in vitro [14]. It comprises colorless, occasionally branched rhizoids, 15 μm in diameter with oblique cross-walls, and an upright, somewhat undulating chloronematal filament system with relatively frequent anastomosis [13,14]. By contrast, the sporophyte is disproportionately large and can be from 7 to 25 mm long [3].

Because the minute gametophyte of *B. viridis* is reputedly impossible to spot in the field, all previous studies have been based on the sporophyte stage [3,4,5]. However, one recent study has described protonemal gemmae (i.e., asexual propagules) in *B. viridis* [13]. These gemmae are multicellular, obloid, with a warty ornamentation, and their size varies between 40 μm and 70 μm. They tend to form brownish, large and highly distinctive aggregations that are relatively easy to locate in the field (see Material and Methods section). 

By conducting non-systematic searches all over France, far beyond the current known range of the sporophytes of *B. viridis*, the authors were able to find many unexpected protonemal populations, for instance in the Northern Oceanic and Mediterranean regions of France. This led the authors to suspect that the species could be well-established at numerous sites far removed from localities where the sporophytes are known, as previously suggested [14].

In this paper, we investigate the vertical distribution of both stages of *B. viridis* in France (i.e., sterile protonema + sporophyte), and we study the ecology of the protonemal stage from a conservation perspective. Do the protonema and sporophyte share the same ecological requirements at two different scales, namely habitat (forest stand) and microhabitat (decaying deadwood)?

## 2. Results

### 2.1. Distribution of Buxbaumia viridis

**Figure 1:**
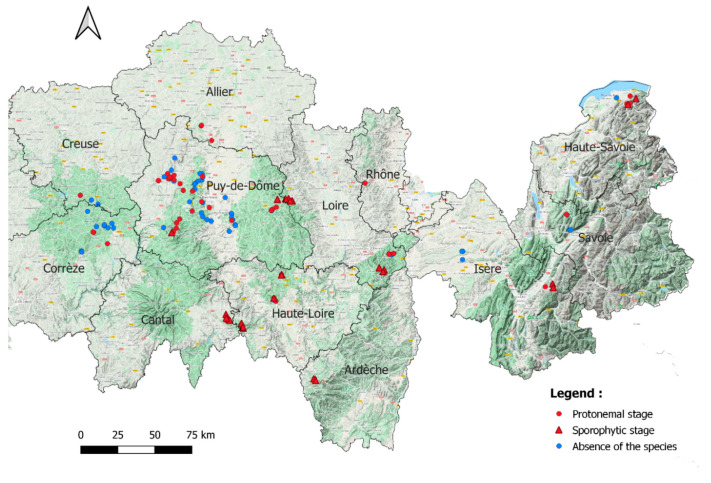
All 137 habitats (forest stands) surveyed. Black lines: French departments limits. © Google Terrain.

*Buxbaumia viridis* was found in 79 forests spread over the 12 French departments (Figure 1). In 50 of them the protonemal stage was found. Sporophyte stage was found in 29 of them, which was always accompanied by gemmae. 

### 2.2. Habitat

Definition of habitat is given in Materials and Methods section. The altitudinal distribution of the protonema localities ranged from 421 m to 1452 m a.s.l (Table 1a). Sporophytes were observed at elevations between 840 and 1452 m a.s.l. More than half of the protonema localities were on north- (27%), northeast- (16%) or northwest-facing (10%) slopes. Of coniferous forests 74, of mixed 78, and of broad-leaved forests 15% contained *B. viridis* (Table 1b). Only protonemal colonies were found in broad-leaved forests. Coniferous forests were dominated by *Pseudotsuga menziesii*, *Abies alba* or *Picea abies*. Mixed forests often consisted of *Fagus sylvatica* associated with *P. menziesii* or *P. abies*. Broad-leaved forests were dominated by *F. sylvatica* or *Acer platanoides*.

**Table 1:** Description of the set of environmental quantitative variables (a) and qualitative variable (b) of all stands surveyed (137), stands containing *Buxbaumia viridis* (sporophyte + sterile protonema) (79) and stands containing sporophytes (29). No: number.

(**a**)				
Variable		Mean ± SD	Min	Max
Elevation (m a.s.l)	All stands	869 ± 273	313	1452
	with *B. viridis*	946 ± 276	421	1452
	with sporophytes	1142 ± 135	840	1452
Slope (degrees)	All stands	17 ± 13	0	50
	With *B. viridis*	18 ± 14	0	50
	With sporophytes	17 ± 14	2	45
Distance to the nearest watercourse (m)	All stands	366 ± 593	8	3330
	With *B. viridis*	279 ± 418	8	3190
	With sporophytes	224 ± 222	10	1156
Deadwood surface (%)	All stands	14 ± 7	3	33
	With *B. viridis*	18 ± 6	3	33
	With sporophytes	19 ± 6	5	33
Northness	All stands	0.29 ± 0.67	−1	1
	With *B. viridis*	0.40 ± 0.61	−1	1
	With sporophytes	0.55 ± 0.48	−0.71	1
**(b)**				
**Forest Type**		**No** ***All stands***	**No** ***with B. viridis***	**No** ***with sporophytes***
Broad-leaved		40	6	0
Mixed		27	21	3
Coniferous		70	52	26
**Total**		137	79	29

**Figure 2:**
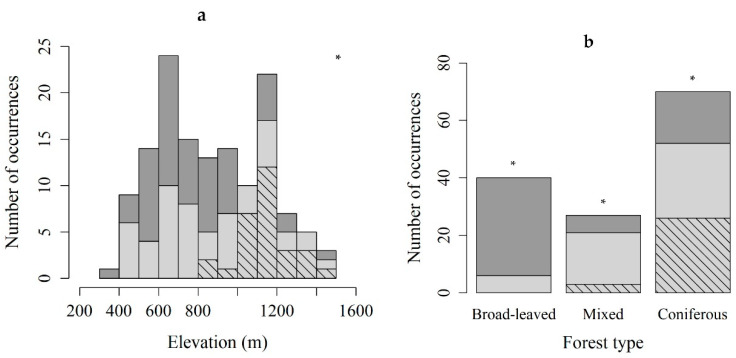
The number of occurrences of the protonemal stage of *Buxbaumia viridis* (light gray) and the sporophyte stage (hatched) classified by elevation (**a**) and forest type (**b**). Dark gray: total stands surveyed. The two variables were significantly different between habitats with sporophytes, and those with protonemal stage only (Kruskal–Wallis chi-squared = 9.7036, df = 1, *p* < 0.05 for elevation, and χ2 test, *p* < 0.05 and absolute residual value >2 for forest type); * *p* ≤ 0.05.

Elevation (Figure 2a) and forest type (Figure 2b) were the only variables differing significantly between habitats with sporophytes, and those with the protonemal stage only.

The occurrence of *B. viridis* is best explained by the extent of deadwood surface and the forest type (Table 2a), and sporophyte occurrence is best explained by elevation, the extent of deadwood surface and Northness (Table 2b). The probability of finding *B. viridis* increases in mixed and coniferous forests compared to broad-leaved forests and increases with increasing extent of deadwood surface. The probability of finding the sporophyte stage increases with increasing extent of deadwood surface, greater elevation, and a northward aspect.

### 2.3. Microhabitat

A definition of microhabitat is given in Materials and Methods section. A total of 275 microhabitats were sampled, and 217 contained *B. viridis* (sterile protonema + sporophyte). Gemmae occupancy had an average value of 30 ± 23 cm² (min = 3, max = 135). Sporophytes were found in 61 microhabitats with an average number of 7 ± 6 sporophytes (min = 1, max = 34). Most of the microhabitats were logs because they were more numerous than stumps.

The mean microhabitat diameter where *B. viridis* (sterile protonema + sporophyte) occurred was 19.0 ± 11.8 cm (Table 3a). Canopy opening was on average 11 ± 4.6%, with a minimum of 2.5% for microhabitats containing *B. viridis*. It was on average 12.8 ± 4.3% for microhabitats containing sporophytes, and never went below 5.5% (Table 3a). Most sporophytes developed on wood with a decomposition stage of 4 and 5, and only 3% on wood with a decomposition stage of 3, whereas gemmae were found a little more on the latter (14%) (Table 3b). The species was never found on wood with a decomposition stage of 2.

The main co-occurring species were *Dicranum scoparium*, which was present on more than half of the microhabitats containing *B. viridis* (sterile protonema + sporophyte) (57%), followed by *Hypnum andoi* (43%), *Hypnum jutlandicum* (41%), *Herzogiella seligeri* (37%), species of the genus *Cephalozia* s.l. (30%), and *Brachythecium rutabulum* (25%).

**Figure 3:**
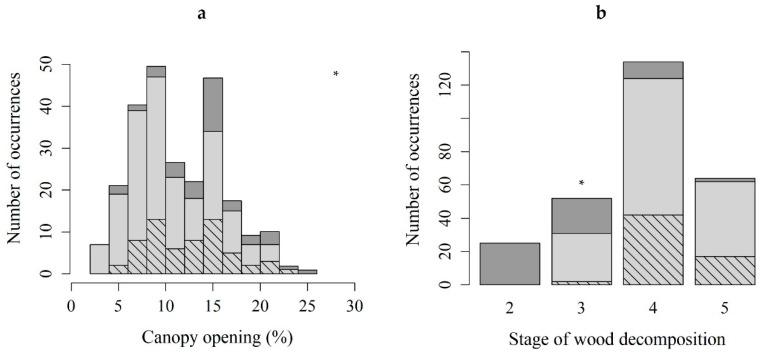
The number of occurrences of the protonemal stage of *Buxbaumia viridis* (light gray) and the sporophyte stage (hatched) classified by canopy opening (**a**) and stage of wood decomposition (**b**). Dark gray: total stands surveyed. The two variables were significantly different between microhabitats with sporophytes, and those with protonemal stage only (Kruskal–Wallis chi-squared = 31.254, df = 1, *p*-value < 0.05 for canopy opening, and χ2 test, *p* < 0.05 and absolute residual value > 2 for stage of wood decomposition); * *p* ≤ 0.05.

Only canopy opening (Figure 3a) and the stage of wood decomposition (Figure 3b) differed significantly between microhabitats with sporophytes, and those with protonemal stage only.

Gemmae occupancy was best explained by decomposition stages 3, 4, and 5 and canopy opening (Table 4a). Gemmae occupancy increased with the stages of decomposition (3, 4, 5) and slightly with reduced canopy opening. The number of sporophytes was best explained by decomposition stages 4 and 5 and canopy opening (Table 4b). It increased with advanced stages of decomposition (4 and 5) and with increased canopy opening.

## 3. Discussion

### 3.1. The Independent Protonemal Populations

Our study provides clear evidence of the existence of independent protonemal populations of *B. viridis* and reveals that the species is certainly much more widespread in France than previously realized. Gemmae were found more often than sporophytes, which confirms that the currently national known range of *B. viridis* is obviously and widely underestimated.

*B. viridis* is apparently a unique case in bryophytes, possessing a morphologically distinct, independent, and persistent protonemal stage. In other bryophyte species where persistent protonema are known (*Ephemerum*, *Tetrodontium*, *Pogonatum*), they are apparently never observed independently from sporophytes, but represent a transitory filamentous phase. The independent protonemal stage of B. viridis is facultative in the sense that it may or may not grow separately from sporophytes, as in the fern species *Pleurosoriopsis makinoi* [15]. As suggested by Duckett et al. [14], it is also similar to another fern, *Vandenboschia speciosa*, which occurs as perennial, gemmiferous gametophytes that have been found in many sites far from localities where the sporophytes are known [16].

Generally, species inhabiting small and transient microhabitats rely more on gemmae formation to persist than species of larger and stable microhabitats [17]. The optimal substrate for *B. viridis* is estimated to last for a few decades on logs, depending on tree species, size (the smaller fragments lasting the shortest time), and humidity [4,18]. Usually spores disperse over long distances by wind, and gemmae disperse predominantly over short distances [2,19,20]. Our results suggest that gemmae are probably more efficiently dispersed than realized. Pohjamo et al. [21] and Rumsey et al. [22] also assumed that asexual propagules as well as spores may contribute to long-distance dispersal, with reference to *Crossocalyx hellerianus,* and *Vandenboschia speciosa,* respectively. Our field observations of frequently trampled and pecked logs suggest that gemmae could be carried efficiently by mammals or birds [23] over considerable distances, or by other means. Nonetheless, it is not known whether independent protonemal populations originate from the germination of spores or gemmae and this should be studied further. 

### 3.2. Habitat and Microhabitat Comparison between Protonemal and Sporophyte Sites

Deadwood surface was an explanatory variable shared by both the sporophyte and protonemal stages: the two generations need a certain amount of deadwood to occur. However, the other variables in their respective models differ: while forest type (mixed and coniferous) was the other explanatory variable for the protonemal stage, the sporophyte was best explained by Northness and elevation. Our results agree with those of Spitale and Mair [3], who also found Northness and the volume of necromass to be environmental predictors for sporophyte occurrence.

Sporophytes of *B. viridis* were found at a narrower range of elevation in the montane zone, almost exclusively in coniferous forests (90%), and on wood in a more advanced state of decomposition (stages 4 and 5). These results are supported by previous studies (in ferns) which demonstrated that the sporophyte generation has a narrower ecological habitat than the gametophyte [17,18]. The wood of coniferous trees (*Abies* or *Picea*) has lower pH values in comparison to broad-leaved trees (*Fagus*) [24], which might seem favorable for the development of *B. viridis* sporophytes [25]. Indeed, the study of Goia and Gafta [26] demonstrated a negative correlation between beech wood and the occurrence of *B. viridis* sporophytes. Another explanation of the supposed affiliation of *B. viridis* to coniferous forests could be different management methods applied between both types of forest [4,27]. Even though we found no significant correlation between deadwood surface and forest type, the average deadwood surface area was lower in broad-leaved forests than in mixed and coniferous forests. In 54% of broad-leaved forests, 10% or less of their surface area was covered by deadwood. Müller et al. [27] also found lower numbers of trunks and stumps in broad-leaved forests.

Elevation is an indirect variable influencing moisture, temperature, precipitation, and solar irradiance [28], thus pointing towards a strong relationship between sporophyte development and microclimatic variables. A detailed climatic study would certainly cast some light on this most relevant issue. Even though no significant correlation was found between the occurrence of *B. viridis* and the distance to the nearest watercourse, moisture can be provided by other variables such as rainfall [3], or microtopography. Northness, which was an explanatory variable for the occurrence of sporophytes, could also provide cooler conditions and greater humidity. The late stages of decomposition in wood occupied by *B. viridis* provide more stable humidity [18,29]. In addition, the species was almost always found on small logs and stumps which are directly in contact with the soil, thus enhancing water intake by capillarity and water retention [29]. At microhabitat scale, the protonemal stage can become established on less decomposed wood (stage 3 versus 4–5 for sporophyte stage) that is more prone to desiccation.

Interestingly, another difference between the sporophyte and protonemal generations is their relation to canopy openness. The probability of finding a high number of sporophytes increases as canopy opening increases, whereas gemmae occupancy follows a reverse trend and increases as canopy opening decreases. We assume that the species occurs frequently as protonema only, and that a slight opening of the canopy could be one of the factors initiating the development of sporophytes. Observations of sporophytes in *B. viridis* are mostly sporadic and scarce [9,30,31], and they could be explained by the generally suboptimal availability of light at forest scale. Our results need to be further investigated as the microtopography of the microhabitat could affect light availability, and thus response to canopy opening [32]. Additionally, shadier localities could favor the development of shade-loving bryophyte species that could outcompete *B. viridis*.

The protonemal stage may provide a biological explanation of observations of *B. viridis* growing on unusual substrates, e.g., on humus [4,7,33], on a disintegrated *Sphagnum* hummock [34], on bark at the base of trees [35], and at very low elevations [7,33]. Protonemal populations may occasionally differentiate into sporophytes as a consequence of temporarily favorable ecological parameters (decreased temperature, high precipitation, local opening of the canopy, etc.). The distribution and rarity of male plants could also account for relative rarity of the sporophyte stage compared with gametophytic one but this has not been addressed here. 

### 3.3. Conservation Implications

In France, and probably at a European scale also, the range of the sporophyte stage of *B. viridis* is currently well-known, but the same is not true for the protonemal stage, whose distribution remains to be clarified. The conservation status of the species is likely to change in coming years, with increasing knowledge of the protonemal stage.

*B. viridis* is often associated with old-growth forests as they tend to be more humid, and to contain larger amounts of coarse wood debris [36]. However, over recent years, increasing doubts have been raised about the status of *B. viridis* as a characteristic species of ancient or natural forests [5,9]. Published studies and our own observations have demonstrated the occurrence of the species in strongly managed forests [5,9], often in young Douglas-fir or spruce plantations.

*B. viridis* is sometimes considered as an umbrella species (i.e., a species whose conservation entails the conservation of notable co-occurring species), and sometimes associated with rare species such as *Buxbaumia aphylla* [33]. It would be worth reconsidering this proposition, as we have consistently found *B. viridis* in assemblages of mundane species.

## 4. Materials and Methods 

### 4.1. Practical Recognition of Buxbaumia viridis Non-Sporophytic Stage

**Figure 4:**
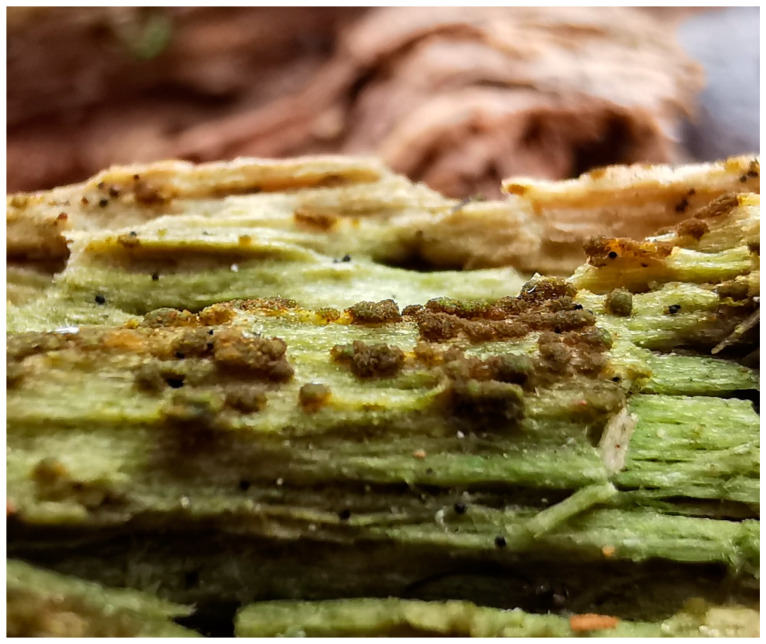
Gametophytic stage of *Buxbaumia viridis* growing on wood-fibers.

**Figure 5:**
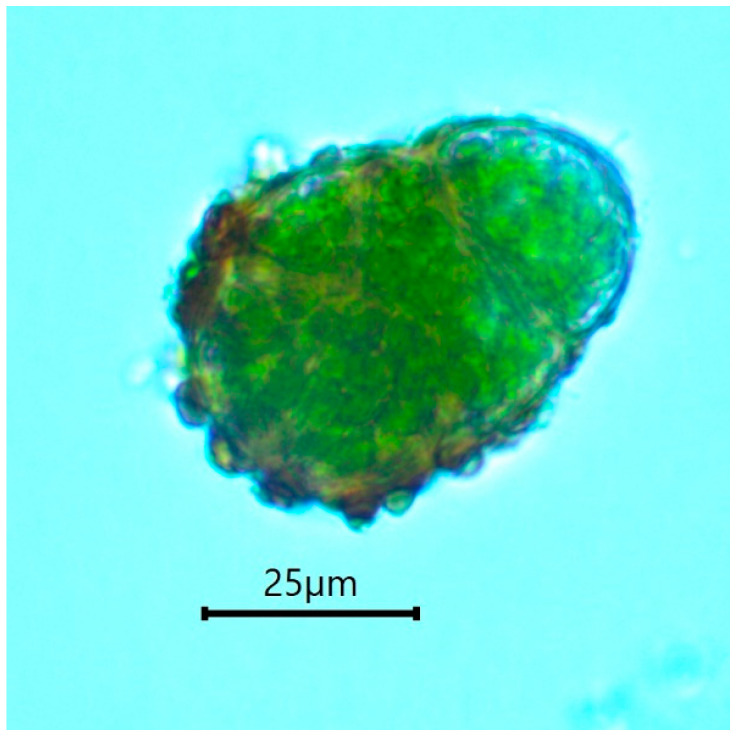
*Buxbaumia viridis* gemma.

**Figure 6:**
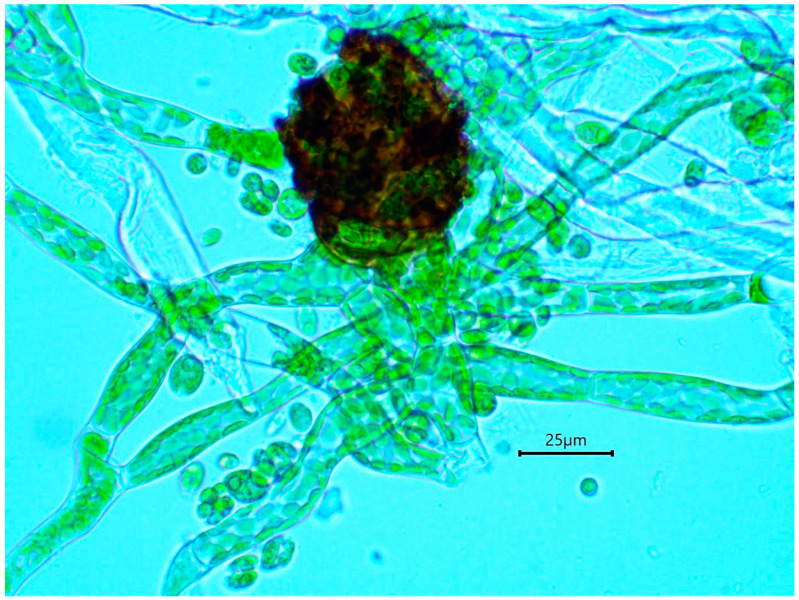
Germinating gemma of *Buxbaumia viridis*, attached to protonema (note anastomosis on the left).

The gametophytic stage of *B. viridis* can be recognized in the field (Figure 4) by the combination of a whitish-grey film immersed in wood fibers (protonema) and discrete chocolate-brown granular masses of protonemal gemmae. A confirmation is provided by a microscopic examination, which shows characteristically multicellular, obloid and warty gemmae (Figure 5) attached to undulating-anastomosing protonema (Figure 6) [14].

### 4.2. Study Area

This study took place in France in 12 departments from the oceanic Limousin (Corrèze, Creuse) to the alpine regions (Isère, Haute-Savoie, Savoie), via the Massif Central (Allier, Ardèche, Cantal, Haute-Loire, Loire, Puy-de-Dôme, Rhône).

**Figure 7:**
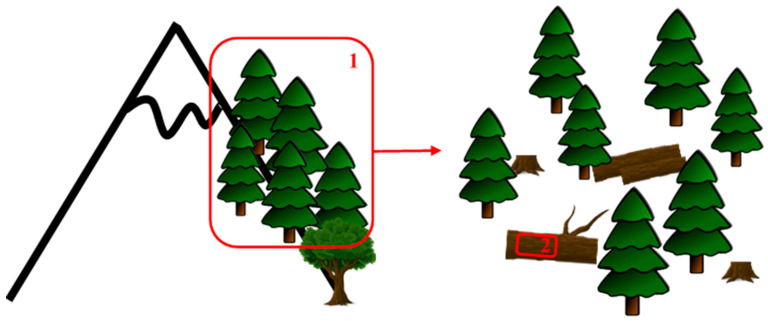
Representation of the two spatial scales used in the study. 1: Habitat (500 m²). 2: Microhabitat (300 cm²).

Forest stands were selected, taking into account accessibility, occurrence of sufficient amount of dead wood, and representativity. Sufficient amount of dead wood corresponds to ≥ 3% of soil surface covered by dead wood. The work was undertaken at two spatial scales: habitat and microhabitat (Figure 7). 

### 4.3. Habitat Scale

Habitat was defined by means of forest stands, each covering 500 m² and encompassing an ecologically homogeneous forest zone, thus excluding topographic intrusions such as screes, rivulets etc. 137 habitat stands were selected in order to capture a relatively large array of environmental factors. A similar sampling time was allocated in each of the stands. We recorded GPS coordinates (Garmin, eTrex Vista HCx), forest type (i.e., coniferous, mixed, broad-leaved), dominant tree species (i.e., tree species covering more than 50% of the stand surface) and aspect (using a compass). We visually estimated slope (in degrees), and the area of soil surface occupied by pieces of deadwood with a diameter >5 cm (% deadwood/500 m²), as a proxy for the amount of deadwood. The occurrence of the protonemal stage and sporophytes was recorded. Elevation and distance from the nearest watercourse (as a proxy for humidity) were assessed (in meters) off-site from topographic maps on Geoportail and on Qgis (3.10.5). 

### 4.4. Microhabitat Scale

**Figure 8:**
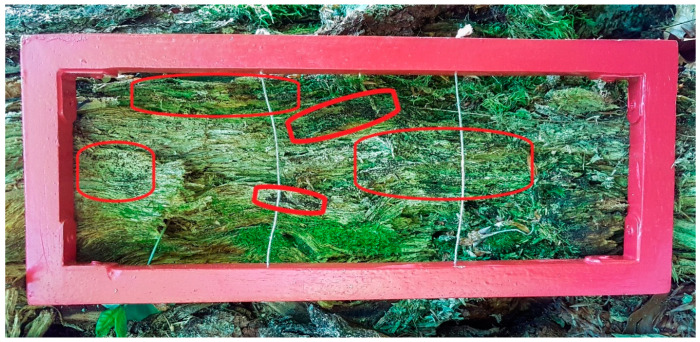
A grid of 30 cm × 10 cm placed on a decaying wood. Groups of *Buxbaumia viridis* gemmae correspond to the small black dots whose main areas are circled in red.

Microhabitat has been restricted to logs and stumps, as a preliminary survey demonstrated that the gametophytic stage was very rare on other substrates (soil, base of trunks etc.). Microhabitat was defined by the presence of favorable woody debris over an area of 300 cm² (Figure 1). 5 microhabitats colonized by *B. viridis* were sampled within each of 55 habitat stands (275 microhabitats recorded). A grid of 30 cm × 10 cm was placed on the woody debris (Figure 8), within which the cover of gemmae (%/300 cm²) and the number of sporophytes (young, old, and eaten) were recorded. The type of deadwood (i.e., log or stump) was noted and its diameter was measured (in centimeters with help of a caliper). Diameter was sometimes impossible to measure when microhabitat was completely crumbled away on soil. Slope was visually estimated (in degrees), and aspect was recorded (using a compass). The degree of decomposition of the wood was determined according to the 5 decay classes proposed [37], going from stage 1 (hard texture, intact bark, round shape, branch present) to stage 5 (soft and powdery texture, no branch, no bark, partly sunken on ground). See Bunnel and Houde Figure 1 for more details [37]. We also noted the dominant type of fungi associated with the decay of the wood (i.e., red or white). Canopy openness was measured using a convex densiometer (Forestry Suppliers, Spherical Crown Densiometer, Model A) (% of sky). Four readings facing North, East, South, and West were taken about a microhabitat and averaged. Finally, we identified co-occurring bryophyte species within the grid, with a visually estimate of their cover (%).

### 4.5. Statistical Analysis

A map of the distribution of *B. viridis* was prepared using Qgis (version 3.10.5).

The aspect variable was converted to ‘Northness’ according to the formula: Northness = cos((aspect in degrees × π)/180), to simplify the analysis [3]. A value close to 1 corresponds to a northward aspect.

Normality was tested using the Shapiro–Wilk test. Since the data do not follow a normal distribution, we used the Kruskal-Wallis non-parametric test to compare the quantitative environmental variables between sporophyte and protonemal sites (habitats and microhabitats). Comparisons of qualitative variables were made using a χ² test. If the *p* value was significant for the χ² test, it was followed by an analysis of the residuals to determine which modalities of the categorical variables were “significant”: residual with absolute value >2 is considered significant [38].

To test the influence of the environment on the occurrence of *B. viridis*, multiple logistic regressions were performed between the binary dependent variables (“presence/absence” of the species (sterile protonema + sporophyte), and “presence/absence” of sporophytes), and the independent environmental variables (forest type, elevation, slope, Northness, deadwood cover on soil, and distance to the nearest watercourse) as predictors. A stepwise selection was made by simultaneously adding and dropping predictors in the model until it led to the best model (i.e., the one with the smallest AIC). The significance of explanatory variables confirming that they play a role in the model was assessed using Wald test (*p* value < 0.05). Multicollinearity between independent variables was tested by checking for the variance inflation factor (VIF). Goodness of fit was estimated using Hosmer–Lemeshow test (*p* value > 0.05). Imperfection detectability (i.e., the inability to detect a species despite its presence) was not considered in our model. This should not be an issue according to Spitale and Mair [3], who demonstrated that the model ignoring imperfection detectability was sufficient to identify the most important environmental factors for species distribution.

Following the same steps as explained above (except for the Hosmer–Lemeshow test), multiple linear regressions were performed to test the influence of microenvironment (using canopy openness, slope of the substrate, bryophyte cover, stage of wood decomposition, and Northness of the substrate as the independent variables) on the dependent variables (1) gemmae occupancy (log-transformed), and (2) number of sporophytes. For the latter, negative binomial regression was preferred to Poisson since the data presented overdispersion. Microhabitat diameter was not included in the models since the analysis concerns gemmae occupancy and number of sporophytes within the 300 cm². All statistical analyses were conducted on R (version 3.5.1).

## 5. Conclusions

Many questions about the ecology and distribution of this species remain to be investigated further. *B. viridis* could be dynamically expanding its range by means of gemmae dispersal, in recent coniferous plantations that are maturing into a favorable condition for a deadwood-dwelling species. In fact, in France, many plantations are nowadays several tens of years of age, the amount of time necessary to begin to accumulate decaying woody pieces. Diachronic monitoring could be conducted to evaluate population dynamics. The sporophytes of *B. viridis* are generally considered weak competitors [5], but this may not be the case for the protonemal stage, as our preliminary observations suggest that it is capable of long-term durability even in closed bryophyte communities (very often behaving as bryo-epiphytic).

Cultivation experiments could answer some biological questions: is the independent protonemal generation of *B. viridis* isolated by a failure to reproduce sexually, or only by limiting environmental variables? Is there a similar independent protonemal stage in other species of the genus *Buxbaumia*?

## Figures and Tables

**Table 2:** Logistic regression results after model simplification using stepwise selection procedure (the model with the smallest AIC (Akaike information criterion) was chosen). The first model (a) tests the relationship between the occurrence of *Buxbaumia viridis* (sterile protonema + sporophyte) as a dependent variable, and environmental variables at habitat scale as independent variables (predictors). Model (b) is similar, except that the dependent variable is the occurrence of sporophytes. Only significant variables were retained in the final model (Wald, *p* < 0.05).

	(a) *Buxbaumia viridis* (Sterile Protonema + Sporophyte)			(b) Sporophyte		
Predictor	β	SE	*p (Wald Test)*	β	SE	*p (Wald Test)*
(Intercept)	−5.282	0.946	**<0.001**	−10.023	1.853	**<0.001**
Mixed forest	3.426	0.790	**<0.001**			
Coniferous forest	3.432	0.699	**<0.001**			
Deadwood surface	0.253	0.049	**<0.001**	0.096	0.043	**0.026**
Elevation				0.007	0.001	**<0.001**
Northness				1.250	0.522	**0.017**
Observations	135			135		
R² Tjur	0.555			0.418		
Hoslem–Lemeshow *p* (Goodness-of-fit test)	0.190			0.854		

**Table 3:** Description of the set of microenvironmental quantitative variables (a) and qualitative variable (b) of all microhabitats sampled (275), microhabitats containing *Buxbaumia viridis* (sterile protonema + sporophyte) (217) and microhabitats containing sporophytes (61). No: number.

(**a**)				
Variable		Mean ± SD	Min	Max
Canopy opening (%)				
	All substrates	12.0 ± 4.7	2.5	24.5
	with *B. viridis*	11.0 ± 4.6	2.5	22.5
	with sporophytes	12.8 ± 4.3	5.5	22.5
Slope (°)				
	All substrates	35 ± 29	0	90
	With *B. viridis*	34 ± 29	0	90
	With sporophytes	32 ± 30	0	90
Northness				
	All substrates	0.28 ± 0.66	−1	1
	With *B. viridis*	0.29 ± 0.66	−1	1
	With sporophytes	0.34 ± 0.61	−0.92	1
Bryophyte cover (%)				
	All substrates	26 ± 21	0	98
	With *B. viridis*	28 ± 21	0	88
	With sporophytes	30 ± 22	0	88
Diameter (cm)				
	All substrates	18.5 ± 11.2	10.0	90.0
	With *B. viridis*	19.0 ± 11.8	10.0	90.0
	With sporophytes	16.4 ± 6.4	10.0	90.0
**(b)**				
**Wood Decomposition**		**No** ***All substrates***	**No** ***with B. viridis***	**No** ***with sporophytes***
	Stage 2	25	0	0
	Stage 3	52	31	2
	Stage 4	134	124	42
	Stage 5	64	62	17
	**Total**	275	217	61

**Table 4:** Results of model regression after model simplification using stepwise selection procedure the (model with the smallest AIC was chosen). The first model (a) tests the relationship between gemmae occupancy as a dependent variable, and environmental variables at microhabitat scale as independent variables. Model (b) is similar, except that the dependent variable is the number of sporophytes, and the model is a negative binomial regression. Only significant variables were retained in the final model (t-test, *p* < 0.05).

	(a) Gemmae Occupancy			(b) Number of Sporophytes		
Predictor	β	SE	*p (t-Test)*	β	SE	*p (t-Test)*
(Intercept)	0.465	0.348	**<0.001**	0.020	1.025	**<0.001**
Decomposition stage 3	1.539	0.317	**<0.001**			
Decomposition stage 4	2.654	0.287	**<0.001**	15.288	0.940	**0.004**
Decomposition stage 5	2.862	0.310	**<0.001**	14.457	0.956	**0.006**
Canopy opening	−0.034	0.017	**0.045**	1.164	0.031	**<0.001**
Observations	275			217		
R² Nagelkerke	0.602			0.487		

## Data Availability

The Data are not publicly available as they are an essential part of further publications under preparation.
